# Supplementary material for: Content-rich biological network constructed by mining PubMed abstracts
Source: BMC Bioinformatics. 2004 Oct 8;5:147. doi: 10.1186/1471-2105-5-147 (PMC528731; doi:10.1186/1471-2105-5-147)
Supplement: Additional File 2 — The original results of the above study (non-essential files are deleted to keep the file size under the limit set by BMC bioinformatics). [file 1471-2105-5-147-S2.bz2 › chilibotAdditionalFile2/dip10/748ID9811664E18130/html/EXBD_TONB.html]

 


 **EXBD** and **TONB** 
  
Found 54 abstracts in PubMed, retrieved 10.  
 

 What does Google say? 
 PDF only 
| .edu only 

---

**Interactive relationship** (e.g. stimulation, inhibition, etc)

**Neutral relationship**- The aim of this review is to describe an in vivo assay of the interactions taking place in the Tol Pal or  **TonB**  ExbB  **ExbD**  envelope complexes in the periplasm of Escherichia coli and between them and colicins or g3p protein of filamentous bacteriophages.  Ref: 12423784 Biochimie< MedlineTA>Biochimie,
- ExbB and  **ExbD**  proteins are part of the  **TonB**  dependent energy transduction system and are encoded by the exb operon in Escherichia coli.  Ref: 12193634 J Bacteriol, 2002
- Both ExbB and  **ExbD**  were also required for association of  **TonB**  with the cytoplasmic membrane.  Ref: 12193634 J Bacteriol, 2002
- Specifically, both ExbB and  **ExbD**  were found to be required for  **TonB**  to conformationally respond to proton motive force.  Ref: 12193634 J Bacteriol, 2002

**Non-interactive relationship** (e.g. studied together, co-existance, homology, etc.)

- **TonB**  CysExbB  **ExbD**  D25N was still able to cross link with the FecA DALTV Cys derivatives in a  **tonB**  tolQ background, even though ExbD25N renders the  **TonB**  ExbBD complex nonfunctional V.  Ref: 12618451 J Bacteriol, 2003
- **TonB** , the energy transducer, appears to go through a cycle during energy transduction, with the absence of both ExbB and  **ExbD**  creating blocks at two points i in the inability of  **TonB**  to respond to the cytoplasmic membrane proton motive force and ii in the conversion of  **TonB**  from a high affinity outer membrane association to a high affinity cytoplasmic membrane association.  Ref: 12193634 J Bacteriol, 2002
- Reverse transcription RT PCR, using RNA from organisms grown under iron replete and iron restricted conditions, revealed that  **tonB** , exbB,  **exbD** , tbpB and tbpA are transcribed as a single unit with expression being up regulated in response to iron restriction.  Ref: 12742718 Vet Microbiol, 2003
- PCR approaches allowed the amplification, cloning and sequencing of A. suis  **tonB** , exbB,  **exbD** , tbpB and tbpA homologues.  Ref: 12742718 Vet Microbiol, 2003
- A complex of three membrane spanning proteins  **TonB** , ExbB and  **ExbD**  couples the chemiosmotic potential of the cytoplasmic membrane with siderophore uptake across the outer membrane.  Ref: 12409204 Biochim Biophys Acta, 2002
- In this study, we report the per cell numbers of  **TonB** , ExbB,  **ExbD**  and FepA for cells grown under iron replete and iron limited conditions.  Ref: 11967085 Mol Microbiol, 2002
- The Ton system consisting of the proteins  **TonB** , ExbB and  **ExbD**  is required for colicin and microcin import.  Ref: 12423780 Biochimie< MedlineTA>Biochimie, 2002
- The ratio of these proteins was calculated at one  **TonB**  two  **ExbD**  seven ExbB under all four growth conditions tested.  Ref: 11967085 Mol Microbiol, 2002
- ExbB and  **ExbD**  do not function independently in  **TonB**  dependent energy transduction.  Ref: 12193634 J Bacteriol, 2002
- Quantification of known components of the Escherichia coli  **TonB**  energy transduction system  **TonB** , ExbB,  **ExbD**  and FepA.  Ref: 11967085 Mol Microbiol, 2002
- In gram negative organisms, high affinity transport of iron substrates requires energy transduction to specific outer membrane receptors by the  **TonB**  ExbB  **ExbD**  complex.  Ref: 12562789 J Bacteriol, 2003
- The exbB,  **exbD**  and  **tonB**  genes of the Pasteurella multocida animal pathogen have been cloned by complementation of an Escherichia coli  **tonB**  mutant.  Ref: 12044675 FEMS Microbiol Lett, 2002
- Pasteurella multocida exbB,  **exbD**  and  **tonB**  genes are physically linked but independently transcribed.  Ref: 12044675 FEMS Microbiol Lett, 2002
- The energy is transduced to FecA by the inner membrane complex,  **TonB** , ExbB, and  **ExbD** .  Ref: 12618451 J Bacteriol, 2003
- Crystal structures of two outer membrane transport proteins, FhuA and FepA, and biochemical data reveal strong long range conformational changes of the proteins upon binding of Fe 3 siderophore complexes and in response to energy transfer from the cytoplasmic membrane into the outer membrane via the  **TonB**  ExbB  **ExbD**  protein complex.  Ref: 11934617 Curr Opin Microbiol, 2002
